# Sensitivity Analysis of Various Geometries of PCD and Cemented Tungsten Carbide Cutting Tools during the Milling of GFRP Composite

**DOI:** 10.3390/polym14081524

**Published:** 2022-04-09

**Authors:** François Ducobu, Eloïse Mélice, Edouard Rivière-Lorphèvre, Thomas Beuscart, Oihan Aizpuru, Aurélie Granjon, Paulo Flores, Denis Soriano, Mikel Cuesta, Pedro-Jose Arrazola

**Affiliations:** 1 Machine Design and Production Engineering Lab, Research Institute for Science and Material Engineering, University of Mons, Place du Parc 20, 7000 Mons, Belgium; meliceeloise@outlook.fr (E.M.); edouard.rivierelorphevre@umons.ac.be (E.R.-L.); thomas.beuscart@umons.ac.be (T.B.); 2 Mechanical and Manufacturing Department, Faculty of Engineering, Mondragon University, 20500 Mondragon, Spain; dsoriano@mondragon.edu (D.S.); mcuesta@mondragon.edu (M.C.); pjarrazola@mondragon.edu (P.-J.A.); 3 Zubiola S. Coop., 20720 Azkoitia, Spain; oaizpuru@zubiola.es; 4 Sobelcomp SPRL. Rue de l’économie 13, 4431 Loncin, Belgium; granjon.aurelie@sobelcomp.be (A.G.); flores.paulo@sobelcomp.be (P.F.)

**Keywords:** milling, GFRP, cutting tool, tool wear, experiments

## Abstract

Although much research has been carried out in the field of the milling of GFRP (Glass Fibre Reinforced Polymer) composites, the complexity of the process is such that it is still not mastered in many industrial cases. The current work is aimed at studying the influence of three different geometries of PCD (PolyCrystalline Diamond) and cemented tungsten carbide cutting tools during the up-milling of GFRP composites at fixed cutting conditions (*v_c_* = 502 m/min and *v_f_* = 420 mm/min). Delamination, cutting forces and tool wear are compared at the fresh and worn states, and the correlation between the lifespan and the cost of the cutting tool is analysed. The main wearing phase of the tools was performed under the conditions of production in the facilities of a company (Sobelcomp, Loncin, Belgium). The results indicate that the PCD tool with the straight edge, inclined peripheral tooth shape produces the smallest total cutting force and less delamination (shortest and lowest number of delaminated fibres) at both fresh and worn states. Moreover, the grinding ability of PCD makes the cutting tool cost per part lower than for cemented carbide. The PCD tool is therefore the best option to mill GFRP parts.

## 1. Introduction

Composites are formed by two or more constituent materials having different physical or chemical properties [1]. Indeed, they are made of a strong and stiff component, called the reinforcement, which gives strength to the material, and a matrix (base material), which protects and transmits the efforts to the reinforcement elements [2]. When combined, these constituents provide properties that the materials do not have alone, such as better stiffness to weight ratio, strength to weight ratio, corrosion resistance and fatigue resistance. Therefore, the final characteristics of the composite are directly linked to the nature and arrangement of the reinforcement as well as to the type and proportion of the matrix [3]. In many cases, when they are properly selected, composites are able to outperform metals for a specific strength to weight (or stiffness to weight) ratio as well as in terms of fatigue and corrosion resistance, or even in terms of thermal and acoustic insulation [4].

The remarkable structural and mechanical properties of composites make them very versatile materials that can be suited to various performance requirements [1]. Moreover, their benefits compared to metals justify the ever-increasing use of composites in various fields such as aerospace, automotive, construction, shipbuilding, etc., to the point that, nowadays, composites constitute one of the broadest and most important classes of engineering materials [2].

Among all the available composites, Glass Fibre Reinforced Polymer (GFRP) dominates the European composite market. In 2018, GFRP represented 95% of this market [5]. Their good mechanical properties and their cost, which is noticeably lower than other fibres, explain the widespread use of this composite material. Due to this global interest for GFRP composites, a significant amount of research has been carried out to gain a better understanding of their mechanical properties, their fatigue, flexural or tensile behaviour and their machinability regarding various conventional and unconventional processes [6].

However, complexity in GFRP machining is such that it is still not mastered in many industrial cases. Indeed, the composite parts are mostly produced near net shape. Therefore, machining operations such as milling are required to achieve dimensional tolerances, manufacture parts that are difficult to mould, finish the edges of laminated composites, etc. [7]. Due to their anisotropic and non-homogeneous characteristics as well as to the highly abrasive nature of their fibres, especially for GFRP [8], composites are difficult-to-machine materials. High abrasive wear effects on the cutting tools are observed, as well as failures in laminates during the process. For these reasons, defects (such as delamination or burrs) can easily occur, and the properties, but also the quality, of the final product can be reduced [9]. To maximise machining efficiency, to produce a high surface quality and to reduce the number of defects, it is crucial to understand the reasons for the presence of these defects. Moreover, studying the process to obtain adequate cutting conditions for a given cutting tools is also essential.

**Figure 1:**
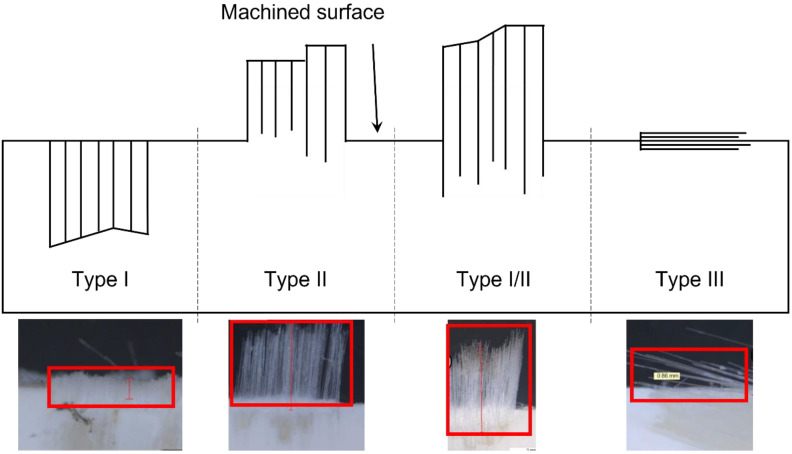
Schematic representation of the four delamination types with examples of the actual defects of each type.

During the milling of composite materials, delamination is one of the most damaging defects that can occur [10]. The origin of delamination is the axial force generated by the helix inclination angle of the cutting tool that cannot be supported by the top and bottom layers of the composite. Four different types of delamination exist (Figure 1) [11,12]. These types and their frequency of occurrence directly depend on the surface orientation of the composite and on the process parameters, such as the cutting speed, the feed rate or the depth of cut [4].

Type I delamination is due to broken and removed fibres below the machined surface. Type II delamination consists of uncut fibres that protrude outward from the machined surface, whereas Type I/II delamination is a combination of both previous types. Type II delamination is caused by the fibres being able to bend or move away from the path of the moving tool and then spring back to their original orientation. Type III delamination represents partially attached fibres or cracks parallel to the machined surface. Both Type I and Type III delamination create loose fibres attached to the machined edge and cause a surface with a fuzzy appearance [12]. Delamination types II and I/II result in a reduction of the quality of the machined composite component. They require manual finishing operations which are labour intensive and significantly increase the cost of the components. They should therefore be avoided as much as possible.

As shown by Geng et al. [13], delamination is a major concern when machining composite materials. Techniques such as rotary ultrasonic elliptical machining were recently introduced to reduce delamination [14]. As previously mentioned, it is primordial to ensure the dimensional accuracy of the workpiece and its mechanical performances. Moreover, in an industrial environment, the aesthetic is also a key factor for the sale of the components. To be compliant to the above-mentioned requirements, the type II and I/II delamination must be avoided. The delamination frequency is estimated by determining the type and size of each occurrence of delaminated fibres on the top and bottom plies of the component. This way, when the frequency of appearance of each delamination type has been counted, it is possible to assess the predominant type for a particular set of cutting parameters [11]. Moreover, Sheikh-Ahmad et al. [11] have determined that, from experience working within the industry, a delamination depth of maximum 1.5 mm might be tolerated.

It has been shown that milling at high spindle speed and low feed rate leads to a decrease of the delamination defects. Furthermore, the use of a high helix angle tool generates a higher surface quality and less tool wear [4]. This illustrates the importance of the choice of the geometry of the cutting tool.

**Figure 2:**
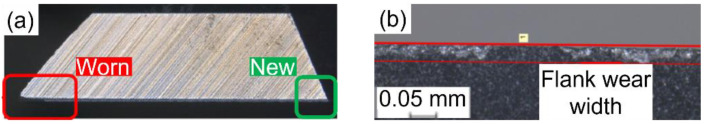
(**a**) Comparison of CER between a worn and a new cutting edge; (**b**) Flank wear on a PCD tool.

When used, and especially for difficult-to-cut materials, cutting tools are wearing, which decreases the efficiency of the cutting tools. This leads to poor material removal and to a reduction of the quality of the machined surface [3]. For materials such as GFRPs, the dominant wear mechanisms are Cutting Edge Rounding (CER) due to the abrasion of the cutting edges (Figure 2a) and the flank wear appearing on the clearance face, creating a slightly curved area (Figure 2b) [4].

Finally, regarding the cutting conditions, the cutting speed should generally be increased, while the feed rate should be decreased for tool wear reduction [4]. To give an order of magnitude, the cutting speed during the milling of fibre-reinforced polymers with a PolyCrystalline Diamond (PCD) tool is generally between 200 m/min and 500 m/min when the feed per revolution (the product of feed per tooth by the number of teeth) is between 0.02 mm and 0.06 mm [15,16].

This paper aims to present the experimental work performed for comparing different cutting tools according to their materials and geometries for the up-milling of a GFRP composite workpiece at fixed cutting conditions. The final objective is to determine which cutting tool provides the best results in terms of delamination and cutting forces. Moreover, a comparison of the performances of the tools at the fresh and at the worn states is provided to analyse the correlation between the tool life and the cost of the tool regarding its material. The innovative approach of this study is that most of the experimental work has been carried out in the facilities of an industrial company, Sobelcomp (Loncin, Belgium), and therefore, under the conditions of production. The results will be analysed in two separated phases corresponding, respectively, to the data collected at the fresh and at the worn states.

## 2. Methodology

This section presents the general process set up to achieve the objectives presented in the introduction. Details considered for the analysis of the delamination, the cutting forces and the tool wear will also be presented.

### 2.1. General Process

The experimental procedure is divided into three main steps. Firstly, the fresh state of each tool is set. Pictures of the cutting tools are taken, and cutting tests with the fresh tools are carried out on GFRP coupons. The two tools that give the best results in terms of cutting forces and delamination are selected to be worn in industrial conditions (step two). These cutting tools were sent to the company where they were used until the occurrence of unacceptable defects on the workpiece. Finally, the same cutting tests as in the first step are performed on the two worn tools to obtain an adequate state of comparison. Pictures of the worn tools are taken, and the results can be analysed. The general methodology is summarised as follows:

- Phase 1: Tests at the fresh state of the cutting tools and selection of the best tools.
- Phase 2: Wear of the best cutting tools at the company under industrial conditions.
- Phase 3: Tests at the worn state of the cutting tools and comparison with the fresh state.

**Figure 3:**
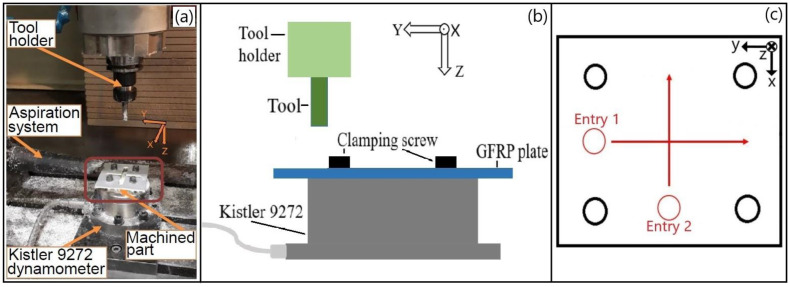
(**a**) Experimental setup; (**b**) Schematic representation of the clamping system of the GFRP coupon on the dynamometer and; (**c**) Tool path.

Cutting tests in Phase 1 and Phase 3 are performed without coolant (dry) on a 5-axis CNC machine (LAGUN Fagor 8070 CNC, Mondragon, Spain) with a 15 kW spindle. As the dust of the GFRP is toxic for the operator, an aspiration system is installed (Figure 3a). To record the cutting forces, a dynamometer (Kistler 9272, Winterthur, Switzerland) is fixed on the table of the machine, as illustrated in Figure 3a. The measured signal (sampling frequency of 25 kHz) is then amplified and filtered (low-pass filter with a cut-off frequency of 5 kHz). The experimental tests are carried out on several coupons in which four M8 through-holes separated by approximately 45 mm are drilled to allow the clamping of the workpiece on the dynamometer. The assembly is schematically illustrated in Figure 3b. This way, no significant vibrations are noted. Therefore, the setup rigidity is considered as suitable for the milling process.

Each tool milled a cross on the GFRP coupon, as illustrated in Figure 3c. Each operation was carried out twice for each tool. The results that will be presented correspond to an average between the two cutting tests and this, for each cutting tool. The cutting conditions (cutting speed *v_c_*, feed rate *v_f_*) are fixed and are detailed in Table 1.

**Figure 4:**
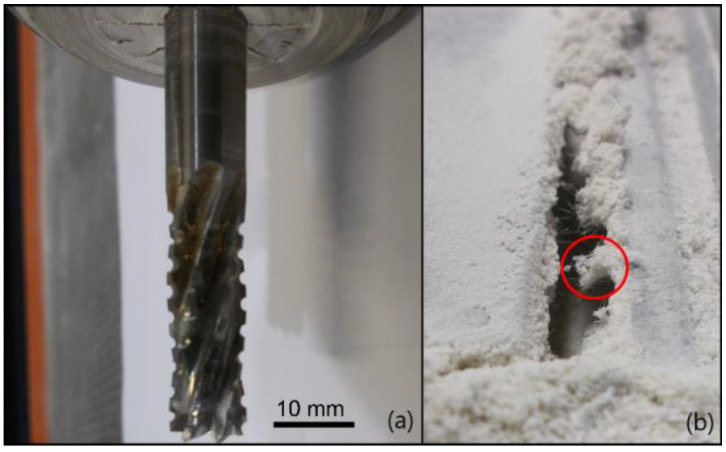
Sobelcomp setup: (**a**) Experimental setup with the T2 cutting tool; (**b**) Closeup on a workpiece (example of delamination under the composite dust highlighted).

Cutting tests in Phase 2 are performed on the same GFRP composite material. They were carried out on a 6-axis robot (Kuka F-85600, Augsburg, Germany) without coolant (dry). When the machined workpiece was unsatisfactory in term of quality (assessment by the operator), the cutting tool was changed. The experimental set-up is partially illustrated in Figure 4. One of the cutting tools, T2, and the GFRP workpiece material at an unacceptable state are visible, respectively, in Figure 4a,b.

The characteristics of the GFRP coupons are given in Table 2.

**Figure 5:**
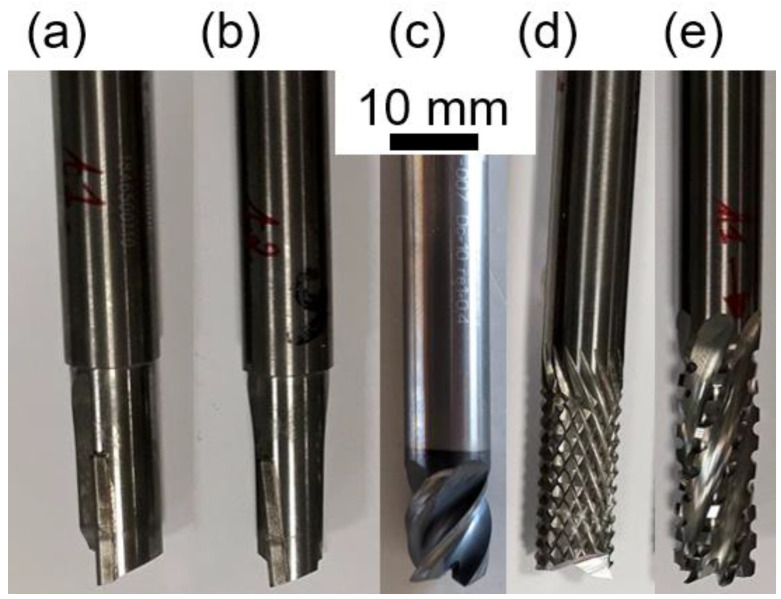
Some tool geometries for milling composites: (**a**) straight flute, (**b**) straight helical flute, (**c**) helical tool, (**d**) burr tool (pyramidal tooth shape) and (**e**) fluted burr tool or satcut tool (trapezoidal tooth shape).

To mill GFRPs, several materials and geometries of cutting tools can be considered. For their resistance to the abrasiveness of glass fibres, PCD or cemented tungsten carbide are preferred. As for their geometries, Figure 5 represents some cutting tools that can be used to mill fibre-reinforced polymers.

**Figure 6:**
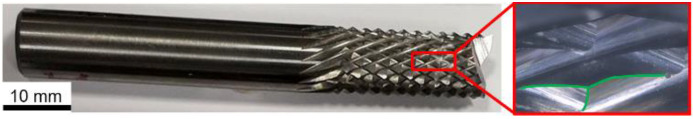
Illustration of a tooth of a burr tool—Periphery tooth highlighted.

The geometry of the burr tool is special: it has diamond shaped teeth due to intersecting up and down helixes [4] (Figure 6). The first one is along the cutting direction and the second one is along the tool axis [17].

The cutting tools were provided by the company Zubiola (Azkoitia, Spain). Table 3 gives the characteristics of the three selected cutting tools used for this study, specifically, the material, diameter, number of end-mill teeth and peripheral tooth shape as well as a picture illustrating the geometry of the tools. These tools exhibit unconventional geometries:

- T1: Cemented carbide burr tool.
- T2: Cemented carbide satcut tool with six end-mill teeth from which six flutes go down the tool axis.
- T3: PCD tool with a slight inclination of the straight secondary cutting edges.

The higher hardness of PCD compared to cemented carbide should provide a better resistance to wear when machining highly abrasive materials such as glass fibres. At constant feed rate, a smaller number of teeth implies a higher feed per tooth, leading to a higher force per tooth and potentially a greater tool wear. T1 combines cemented carbide and a small number of teeth that should results in lower performances as compared to the two other tools. It is more difficult to predict the performance of T2 and T3 as they exhibit opposed characteristics (softer material and larger number of teeth versus harder material and smaller number of teeth).

### 2.2. Delamination

One particularity of composite materials is that, regarding their manufacturing process, the fibres are not arranged in the same way in the resin, preventing assessment of the damages in a reliable way. Indeed, the cutting tools statically cut the same number of fibres. However, due to the random distribution of fibres, the resin can sometimes cover specific fibres, making them less visible. To remain objective, the following criterion is used for this study: the highest determined number of delaminated fibres of a tool is taken as the maximum value (100%). The number of delaminated fibres of the other tools are weighted on this value.

As highlighted in the introduction, delamination types I/II and II lead to poor dimensions and must be avoided. Therefore, the frequency of the appearance of these two types will be a determining criterion. Moreover, the other aspect to assess the efficiency of a cutting tool is the length (depth) of the uncut or delaminated fibres. Indeed, according to Sheikh-Ahmad et al. [11], a delamination depth of maximum 1.5 mm might be accepted. The measurement of the delamination as well as of the tool wear were achieved with a Leica Z16 APO microscope (Wetzlar, Germany), and the processing was performed with the associated LAS Core software (Wetzlar, Germany).

### 2.3. Cutting Forces

Cutting forces may be linked to observed delaminations during milling. The Kistler 9272 dynamometer (Winterthur, Switzerland) recorded, during the entirety of each experimental tests, the maximal forces in the three directions *F_x,max_*, *F_y,max_* and *F_z,max_*. For the analysis, the total cutting force, *F_tot_*, is calculated based on the equation:

(1) $$F_{tot}=\sqrt{F_{x,max}^{2}+F_{y,max}^{2}+F_{z,max}^{2}}$$

Knowing that the machining operation is carried out two times, *F_i,max_* (with *i* = *x*, *y*, *z*) corresponds to the mean value of the maximal forces acquired during the first and the second experimental tests for each tool. The maximal thrust force generated during the milling corresponds is *F_z,max_*, and this will also be analysed. This uncommon maximum Z axis force in the milling process is due to the plunge entry to the material before moving along the X or Y axis.

### 2.4. Tool Life

The measurement of the tool wear was also performed with a Leica Z16 APO microscope (Wetzlar, Germany). The considered tools have different cutting edge geometries (T1 has pyramidal tooth shape, T2 has trapezoidal tooth shape and T3 has straight edges). To assess the tool wear, views of the faces that present wear patterns will be provided. Moreover, to obtain a clear state of comparison, the same faces will be illustrated at their fresh state. The cutting distance that the tools were able to machine before being considered as worn will also be analysed to compare their lifespan regarding their material and cost.

## 3. Experimental Results

Once the experimental procedure is achieved, the following output data are available at the fresh state: delamination count and cutting forces. At the worn state, the delamination, the cutting forces and also the tool wear and the cutting length can be examined.

### 3.1. Delamination

**Figure 7:**
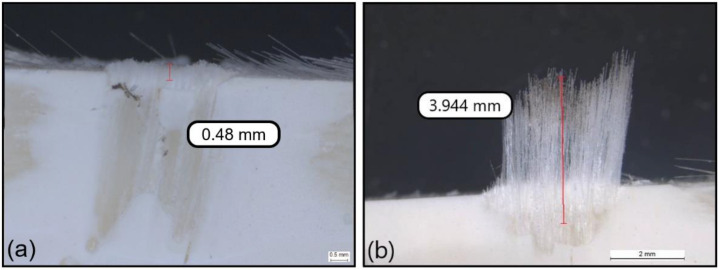
Illustration of the delamination depth: (**a**) type I and (**b**) type I/II caused by T3 at the fresh state.

**Figure 8:**
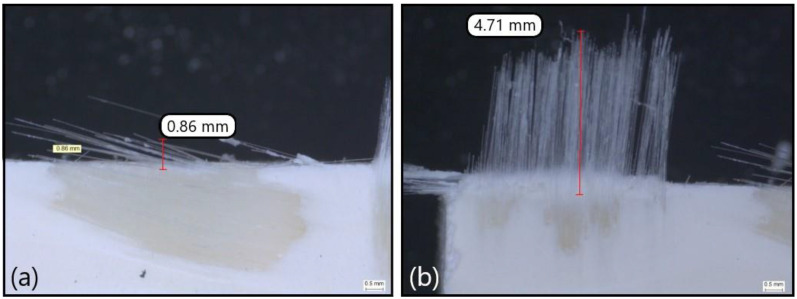
Illustration of the delamination depth: (**a**) type III and (**b**) type II caused by T3 at the worn state.

As highlighted in the introduction, the delamination types I/II and II must be avoided, and a delamination depth of 1.5 mm might be accepted. Figure 7 and Figure 8 show examples of pictures taken with the microscope to assess delamination on GFRP coupons after milling. Each picture illustrates one specific delamination pattern: type I, type I/II, type III and type II.

#### 3.1.1. Fresh State

**Figure 9:**
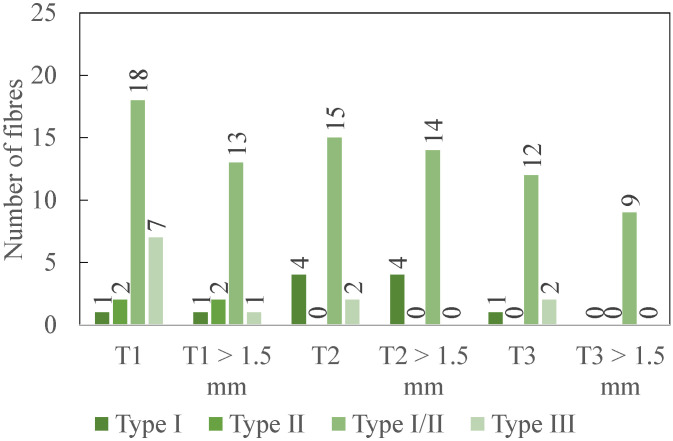
Experimental results: number of delaminated fibres (total and longer than 1.5 mm) after the up milling for 240 mm (2 coupons) at the fresh state-average values for one coupon.

The various lengths of the delaminated fibres are detailed in Figure 9. For each type of delamination, the total number of delaminated fibres is provided. This total number is equal to an average for the delaminated fibres measured on the two GFRP coupons. Only the fibres with a length larger than the threshold of 1.5 mm are considered.

#### 3.1.2. Worn State

**Figure 10:**
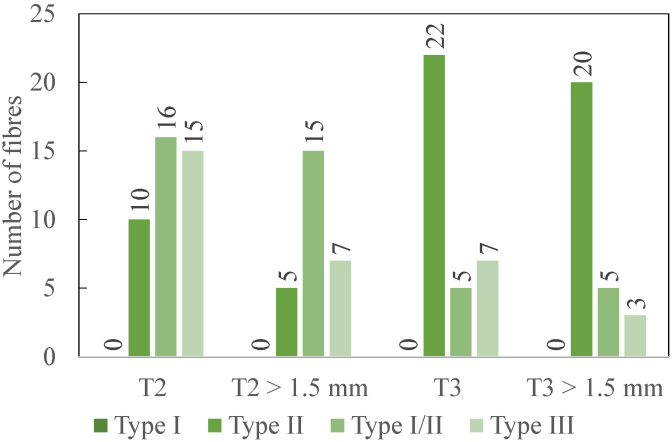
Experimental results: number of delaminated fibres (total and longer than 1.5 mm) after the up milling for 240 mm (2 coupons) for T2 and T3 at the worn state-average values for one coupon.

Figure 10 provides the results regarding the delamination of the two tools (T2 and T3) that have been worn; the same quantities are plotted at the fresh state.

#### 3.1.3. Comparison of the Fresh and Worn States

**Figure 11:**
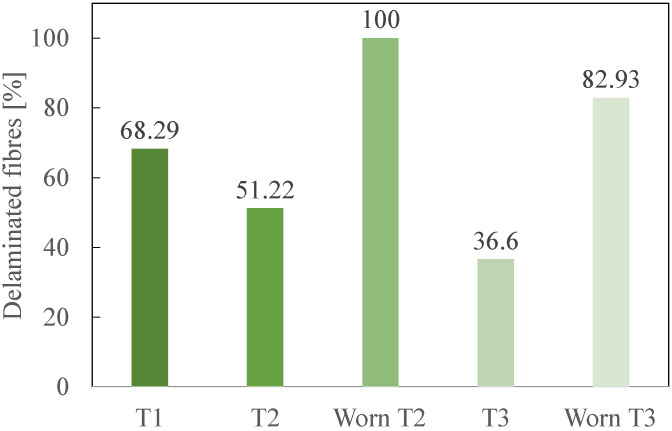
Percentage of total delaminated fibres for each cutting tool.

**Figure 12:**
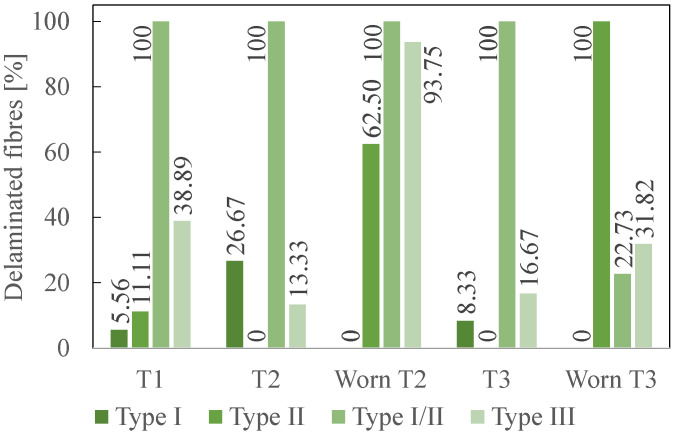
Percentage of delaminated fibres of each delamination type for each cutting tool.

To compare the tools at the two states, Figure 11 illustrates the percentage of delaminated fibres only at the fresh state for T1 and at the fresh, but also at the worn state, for T2 and T3. Figure 12 details the percentages for each delamination types.

### 3.2. Cutting Forces

**Figure 13:**
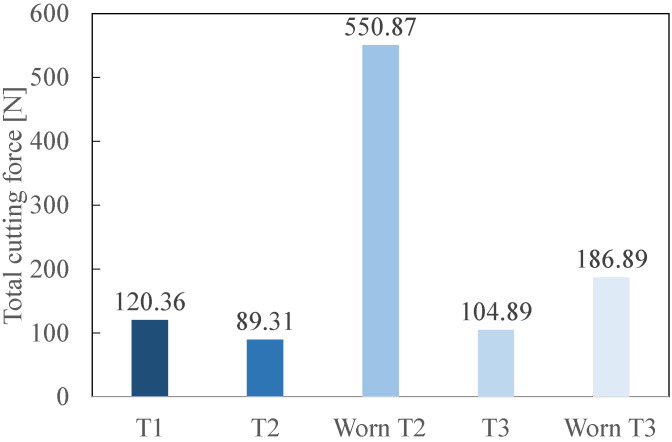
Total Cutting Forces, *F_tot_*, recorded during the milling (fresh and worn cutting tools).

**Figure 14:**
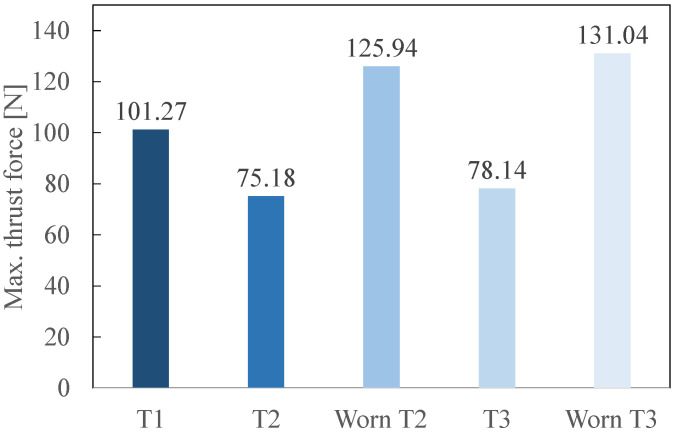
Maximal Thrust Force, *F_z,max_*, recorded when entering the GFRP plate (fresh and worn cutting tools).

Figure 13 gives the total cutting forces for the fresh and worn tools. Figure 14 represents the maximal thrust forces acquired for each tool during the process. This force is maximal when the tool begins its entry into the GFRP composite. As for Figure 13, both fresh and worn states are presented.

### 3.3. Tool Life

Finally, the tool wear that occurred at the end of the machining operation can be analysed. As a reminder, in the first phase (fresh tools), the cutting tools only milled 240 mm of GFRP. This distance is too short for the cutting tools to present any wear damages. To have a true comparison state, only the tools that have been worn (T2 and T3) will be considered. The faces that present damages are observed with a Leica Z16 APO microscope (Wetzlar, Germany) to measure any degradation. The cutting distances that the cutting tools were able to machine in industrial conditions are provided in Table 4.

#### 3.3.1. T2: Cemented Tungsten Carbide Cutting Tool

**Figure 15:**
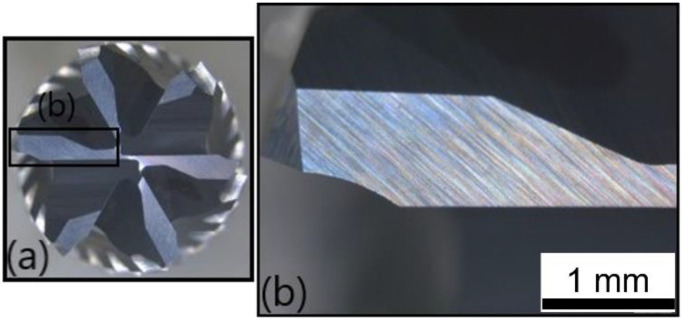
T2: Front view at the fresh state: (**a**) global view of the end-mill and (**b**) zoom on a top tooth clearance face.

**Figure 16:**
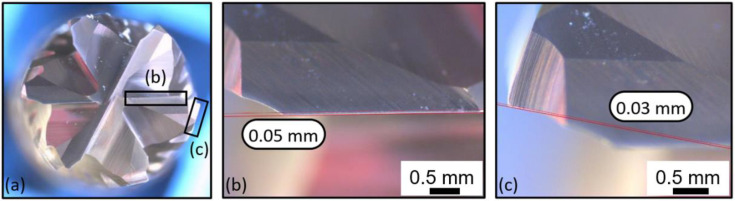
T2: Front view at the worn state: (**a**) global view of the end-mill, (**b**) zoom on a clearance face and (**c**) zoom on the other clearance face.

Figure 15 illustrates the front view of the second cutting tool, T2, at the fresh state. The inspection did not reveal any damages or traces of wear. Figure 16 shows the same front view after the wear of the tool. A flank wear of VB = 0.05 mm was measured on the clearance face (Figure 16b), and a flank wear of VB = 0.03 mm was measured on the other clearance face (Figure 16c).

**Figure 17:**
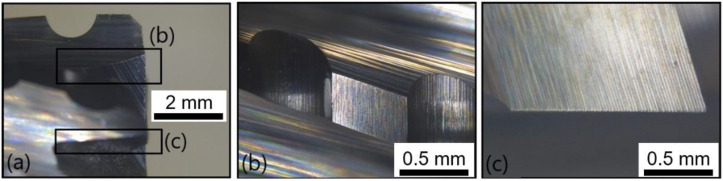
T2: Lateral view at the fresh state: (**a**) global view of the end-mill, (**b**) zoom on a clearance face and (**c**) zoom on another clearance face.

**Figure 18:**
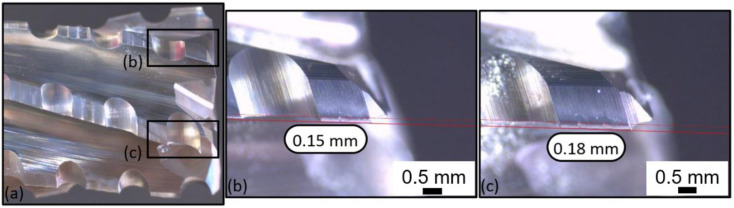
T2: Lateral view at the worn state: (**a**) global view of the end-mill, (**b**) zoom on a clearance face and (**c**) zoom on another clearance face.

Figure 17 provides lateral views of T2 at the fresh state; no wear is noticeable. Figure 18 shows the same lateral view after the wear of the tool. Flank wears of respectively VB = 0.15 mm and VB = 0.18 mm were detected on clearance faces (Figure 18b,c).

#### 3.3.2. T3: PCD Cutting Tool

**Figure 19:**
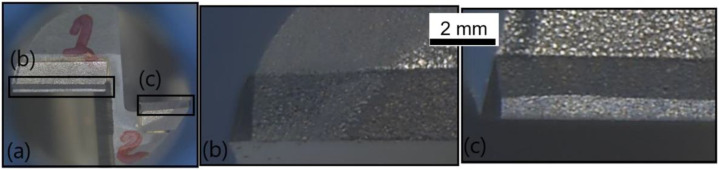
T3: Front view at the fresh state: (**a**) global view of the end-mill, (**b**) zoom on the clearance face of the first cutting edge and (**c**) zoom on the clearance face of the second cutting edge.

**Figure 20:**
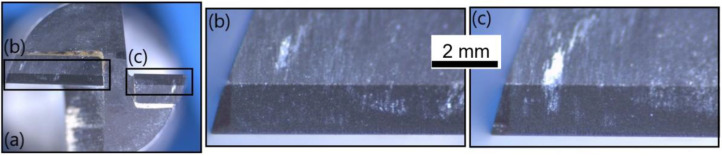
T3: Front view at the worn state: (**a**) global view of the end-mill, (**b**) zoom on the clearance face of the first cutting edge and (**c**) zoom on the clearance face of the second cutting edge.

The following results are available to analyse the tool wear on the PCD cutting tool, T3, at the fresh and at the worn states. Figure 19 provides pictures of the front view of T3 at the fresh state; here again, no wear is observable. The global view of the tool (Figure 19a) and two zooms on the two clearance faces of the two cutting edges (Figure 19b,c) are presented. Figure 20 illustrates the same faces at the worn state, where no flank wear is noticeable. However, some adhesion of the matrix can be noted.

**Figure 21:**
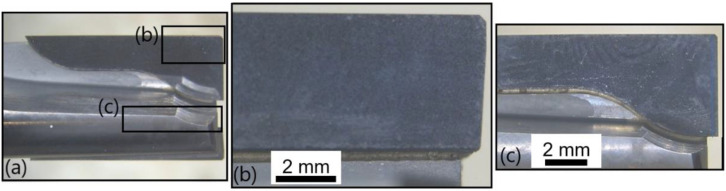
T3: Lateral view at the fresh state: (**a**) global view of the end-mill, (**b**) zoom on the clearance face of the first cutting edge and (**c**) zoom on the clearance face of the second cutting edge.

**Figure 22:**
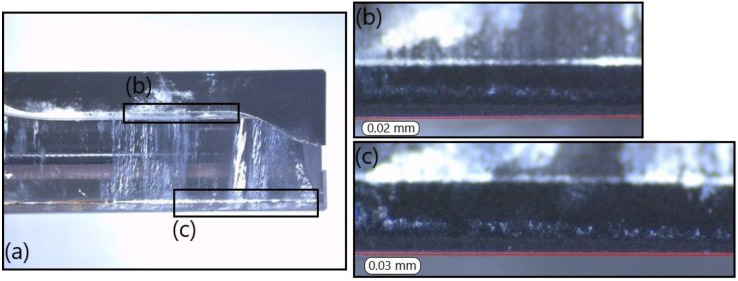
T3: Lateral view at the worn state: (**a**) global view of the end-mill, (**b**) zoom on the clearance face of the first cutting edge and (**c**) zoom on the clearance face of the second cutting edge.

Finally, the lateral face of T3 is analysed at the fresh and at the worn states. Figure 21 provides pictures of the secondary cutting edges at the fresh state. Figure 22 shows the same faces of the tool at the worn state. Flank wears of VB = 0.02 mm and VB = 0.03 mm are noticeable, respectively, on Figure 22b,c). Matrix adhesion is also present on the cutting tool.

## 4. Discussion

### 4.1. Delamination

The results at the fresh state are first analysed. According to the data in Figure 9, the most measured delamination type is I/II with 70.31% of the total number of delaminated fibres (for all types). It is followed by type III (17.19%), type I (9.38%) and finally by type II (3.12%). Types II and I/II are to be avoided as a priority [12]. T1 performance is the worst as it is the only tool that generates delamination type II and the highest number of type I/II delaminated fibres, followed by T2 (83.33%) and T3 (66.67%). Regarding the frequency of appearance of type II and I/II, T3 gives the best results. The same conclusion is reached if the sum of all the delamination types is considered. Indeed, T3 is also the cutting tool that presents the smallest number of delaminated fibres (50%), all types taken together.

The length of the delaminated fibres is then assessed. The longest delaminated fibre created during the milling process had a length of 5.88 mm (type I/II) and was machined by T2. Fibres with a length larger than the acceptable threshold (1.5 mm) should be avoided [11]. This kind of fibre is the most measured for delamination type I/II. Indeed, regarding the other types, there are only a few fibres. T3 stands out from the other tools and provides the best results when the length of the delaminated fibres (type II) is considered. T1 and T2 generate a very similar amount (respectively, 92.86% and 100%) of type II delaminated fibres larger than the threshold. T3 is therefore the best option at the fresh state.

The same analysis is then performed for worn T2 and worn T3. According to the data in Figure 10, the most frequent delamination type is II with 42.67% of the total number of delaminated fibres (for all types). It is followed by type III (29.33%) and type I/II (28%), which show very similar results. Type I is not observed anymore. Compared to the delamination types recorded at the fresh state, the number of type II delamination has highly increased, while type I/II has significantly decreased. Performances of worn T2 and T3 for types I/II and II are opposed. Indeed, worn T2 gives the highest number of delaminated fibres for type I/II, while worn T3 produces only 31.25% of this maximum number. In contrast, worn T3 shows the highest number for type II, while worn T2 generates only half as much delaminated fibres of this type (45.45%).

Worn T3 lead to the longest delaminated fibre, which is 4.71 mm long (type II). This length is smaller than the maximal length recorded at the fresh state, showing an evolution in the action of the tool on the material. The fibres with a length larger than the threshold are the most frequently measured for type II and type I/II. Again, worn T2 gives the highest number of delaminated fibres for type I/II, while worn T3 only produces 20% of this maximum number. In contrast, worn T3 shows the highest number for type II, while worn T2 generates a quarter less delaminated fibres (25%).

A comparison between the fresh and the worn states is carried out based on Figure 11. Its analysis highlights, as expected, that worn cutting tools create more delaminated fibres than fresh ones. Indeed, worn T2 generated approximately twice as much delaminated fibres than fresh T2, while worn T3 created 2.27 times as many fibres as fresh T3. It also clearly shows that T3 performs the best at both fresh and worn states, with approximately 20% less delaminated fibres than T2.

### 4.2. Cutting Forces

The total cutting force and the maximal thrust force are considered. At the fresh state, according to Figure 13, T2 generates the smallest cutting force (89.31 N). This is explained by its geometry. T2 is followed by T3 (104.89 N) and finally T1 (120.36 N). At the fresh state, T2 and T3 show the best results regarding the total cutting force. At the worn state, worn T2 produced a total cutting force drastically larger (550.87 N) than T3. Indeed, its total cutting force is approximately 6 times larger than for its fresh state. Worn T3 clearly gives better results than worn T2 regarding the total cutting force as it only generates 186.89 N.

For the maximal thrust force (Figure 14), fresh T2 and T3 show very similar forces (75.18 N and 78.14 N, respectively), lower than for fresh T1. The values of the thrust force for worn T2 and worn T3 are quite similar; an increase of approximately 50 N is recorded compared to their fresh states.

The increase of the cutting forces can be linked to the number of delaminated fibres. When Figure 11 and Figure 13 are compared, it is observed that an increase of the cutting force leads to a rise in the number of delaminated fibres. Indeed, an increase of approximately 50% was observed in the cases of worn T2 compared to fresh T2 and worn T3 compared to fresh T3. For both the total cutting force and the number of delaminated fibres, T3 outperforms T2 at both fresh and worn states. This shows that the combination of a harder material (PCD instead of cemented carbide) and a smaller number of teeth (2 instead of 6) is best suited to mill GFRP.

### 4.3. Analysis of the Link between the Tool Wear and the Lifespan of the Cutting Tools Regarding Their Materials

In industrial conditions, T2 and T3 tools were able to mill, respectively, 235 m and 729 m of composite material (Table 4) before being replaced due to poor machining results. Table 5 provides a global comparison between T2 and T3 regarding their fresh and worn states, as well as their lifespan and cost. In this table, T2 is considered as the reference tool. This means that its cost (in EUR) and its tool life (in meters) are the references and are equal to 100% (the actual value is not provided for confidentiality reasons).

Firstly, a link between the tool wear and the cutting forces is shown. According to Table 5, a worn cutting tool generates a higher total cutting force than a fresh tool. In addition, less flank wear was measured on worn T3 than on worn T2. Specifically, only two traces of flank wear and some matrix adhesion were observed on worn T3 (VB = 0.02 mm and VB = 0.03 mm) compared to four larger traces on worn T2 (VB = 0.05 mm, VB = 0.03 mm, VB = 0.18 mm and VB = 0.15 mm). This leads to a much higher total cutting force in the case of worn T2. The same observation is valid for delamination. Indeed, the increase of the tool wear, as well as of the cutting forces, lead to a rise in the total number of delaminated fibres. This clearly highlights the better results of T3 compared to T2.

Secondly, the cost of the cutting tools is linked to their lifespan. T3 costs approximately 2.6 times more than T2 but exhibits a tool life 3.1 times larger. Combining cost and lifespan shows that a single T3 is needed to machine the same cutting distance as three T2s. Moreover, T3 can be resharpened at least ten times at a lower cost than a new T2 (Table 5). Resharpening allows an increase in the lifespan of T3 to 34.3 times that of T2. Ultimately, to machine one meter of the same composite material, T3 is 68% less expensive than T2.

The lower machining cost of T3 consolidates the conclusion that it is the best option. Indeed, it leads to less delaminated fibres than T2 and its cost per machined meter is lower. As the purchase cost of T3 is higher than T2, T3 can be seen as a long time run investment.

## 5. Conclusions

In this paper, a comparative analysis of various geometries of PCD and cemented tungsten carbide cutting tools for the milling of GFRP composite material was carried out. The performances of these cutting tools at their fresh and worn states were compared in terms of delamination and cutting forces. The results of this study show that the PCD tool (T3) is the best option by combining less delamination, lower forces and lower overall cost:

- At the fresh state, the straight flute PCD tool (T3) provides the shortest and the lowest number of delaminated fibres at the fresh state and, therefore, the best results when compared to cemented carbide tools (burr tool, T1, and satcut geometry, T2).
- At the worn state, the total number of delaminated fibres increases for both PCD and T2 cemented carbide tools. However, the PCD tool still generates less delamination.
- The total cutting force is the lowest for the cemented carbide tool with more teeth (T2) than the PCD tool (T3) and finally the cemented carbide tool with the same number of teeth as the PCD tool (T1). Wear leads to an increase of the cutting force. The increase for the PCD tool is, however, far smaller than for the T2 cemented carbide tool. The performance of the worn PCD tool is therefore the best thanks to a lower flank wear.
- The higher resistance to wear and the ability to be ground allows T3 (PCD) to machine a length three times longer than T2 (cemented carbide). Therefore, even if T3 is more expensive than T2, the cost per meter of T3 is 68% less expensive than for T3.

## Figures and Tables

**Table 1:** Up milling-Cutting conditions.

*v_c_* (m/min)	*v_f_* (mm/min)	Total Cutting Distance (mm)
502	420	120 per coupon

**Table 2:** Characteristics of the GFRP coupons.

Dimension (mm)	Composition	Fibre Orientation	Density of Reinforcement (g/m^2^)
90 × 90 × 4	76% resin–24% reinforcement (22% glass)	(0°/90°)	800

**Table 3:** Characteristics of the cutting tools used during the milling of the GFRP workpiece.

	T1	T2	T3
Material	Cemented tungsten carbide (uncoated)	Cemented tungsten carbide (uncoated)	PCD
Diameter (mm)	10	10	10
Number of end-mill teeth	2	6	2
Peripheral tooth shape	Pyramidal	Trapezoidal	Straight edge inclined
Illustration	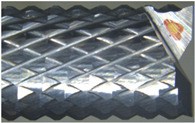	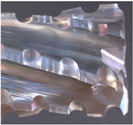	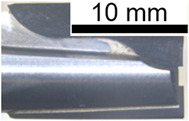

**Table 4:** Total cutting distance for the wear of the cutting tools (Sobelcomp).

	T2	T3
Cutting distance (m)	235	729

**Table 5:** Summary table: tool wear, tool life and tool cost (#: number).

	**Fresh**		**Worn**			
	**Total Cutting Force (N)**	**# Delaminated Fibres**	**Total Cutting Force (N)**	**# Delaminated Fibres**		
T2	89.31	21	550.87	41		
T3	104.89	15	186.89	34		
	**Life and Costs**				**Results**	
	**Tool Cost (%)**	**Tool Life (%)**	**Resharpening Cost (Tool Cost %)**	**# Resharpening Operations**	**Tool Life (%)**	**Tool Cost per Meter (%/m)**
T2	100	100	0	0	100	100
T3	259	310	82	10	3410	32

## Data Availability

Not applicable.

## References

[1] Rajak D.K., Pagar D.D., Kumar R., Pruncu C.I. (2019). Recent Progress of Reinforcement Materials: A Comprehensive Overview of Composite Materials. J. Mater. Res. Technol..

[2] Clyne T.W., Hull D. An Introduction to Composite Materials. https://www.cambridge.org/highereducation/books/an-introduction-to-composite-materials/402C0AAFF04754C676E22D2B1F9080DB.

[3] Bai J. (2013). Advanced Fibre-Reinforced Polymer (FRP) Composites for Structural Applications.

[4] Sheikh-Ahmad J.Y. (2009). Machining of Polymer Composites.

[5] Witten E., Mather V., Sauer M., Kühnel M. (2018). Composites Market Report 2018: Market developments, trends, outlooks and challenges.

[6] Panneerselvam T., Raghuraman S., Kandavel T.K., Mahalingam K. (2020). Evaluation and Analysis of Delamination during Drilling on Sisal-Glass Fibres Reinforced Polymer. Meas. J. Int. Meas. Confed..

[7] Geier N., Davim J.P., Szalay T. (2019). Advanced Cutting Tools and Technologies for Drilling Carbon Fibre Reinforced Polymer (CFRP) Composites: A Review. Compos. Part A Appl. Sci. Manuf..

[8] Cepero-Mejías F., Curiel-Sosa J.L., Blázquez A., Yu T.T., Kerrigan K., Phadnis V.A. (2020). Review of Recent Developments and Induced Damage Assessment in the Modelling of the Machining of Long Fibre Reinforced Polymer Composites. Compos. Struct..

[9] Bayraktar Ş., Turgut Y. (2020). Determination of Delamination in Drilling of Carbon Fiber Reinforced Carbon Matrix Composites/Al 6013-T651 Stacks. Measurement.

[10] Prashanth I.S.N.V.R., Ravi Shankar D.V., Manzoor Hussain M., Chandra Mouli B. (2018). Critical Analysis in Milling of GFRP Composites by Various End Mill Tools. Mater. Today Proc..

[11] Sheikh-Ahmad J.Y., Dhuttargaon M., Cheraghi H. (2017). New Tool Life Criterion for Delamination Free Milling of CFRP. Int. J. Adv. Manuf. Technol..

[12] Colligan K., Ramulu M. (1992). The Effect of Edge Trimming on Composite Surface Plies. Manuf. Rev..

[13] Geng D., Liu Y., Shao Z., Lu Z., Cai J., Li X., Jiang X., Zhang D. (2019). Delamination Formation, Evaluation and Suppression during Drilling of Composite Laminates: A Review. Compos. Struct..

[14] Geng D., Liu Y., Shao Z., Zhang M., Jiang X., Zhang D. (2020). Delamination Formation and Suppression during Rotary Ultrasonic Elliptical Machining of CFRP. Compos. Part B Eng..

[15] Davim J.P. (2009). Machining Composite Materials.

[16] Hintze W., Brügmann F. (2017). Influence of Curved Workpiece Contours on Delamination During End Milling of FRP. Procedia CIRP.

[17] Janardhan P., Sheikh-Ahmad J., Cheraghi H. Edge Trimming of CFRP with Diamond Interlocking Tools. Proceedings of the Aerospace Manufacturing and Automated Fastening Conference and Exhibition.

